# Shifting paradigms: key tensions in putting the person’s narrative central in improving quality of care in Dutch nursing homes

**DOI:** 10.1093/geront/gnag236

**Published:** 2026-10-05

**Authors:** Mirjam Kalisvaart, Lieke Oldenhof, Roland Bal, Anne Margriet Pot

**Affiliations:** Erasmus School of Health Policy and Management, Erasmus University Rotterdam, Rotterdam, The Netherlands; Erasmus School of Health Policy and Management, Erasmus University Rotterdam, Rotterdam, The Netherlands; Erasmus School of Health Policy and Management, Erasmus University Rotterdam, Rotterdam, The Netherlands; Erasmus School of Health Policy and Management, Erasmus University Rotterdam, Rotterdam, The Netherlands; Dutch Health and Youth Care Inspectorate, Utrecht, The Netherlands; Optentia, North-West University, Vanderbijlpark, South Africa

**Keywords:** Nursing home, Quality improvement, Story, Cultural change, Accountability

## Abstract

**Background and Objectives:**

Narrative approaches to quality improvement are increasingly adopted by long-term care organizations to gain insight into quality from the perspectives of older persons. However, it remains unclear how organizations experience the organizational challenges of embedding these approaches in new quality improvement routines and policies. This study aims to investigate what tensions arise when organizing and embedding narrative approaches and how organizations respond to these tensions.

**Research Design and Methods:**

An exploratory qualitative multiple-method design consisting of observations, semi-structured interviews, and document analysis was used. Data were collected in Dutch nursing homes between February 2024 and June 2025. To gain in-depth and broader insights, organizations were observed for a longer period (*n* = 2) and interviews were conducted with other organizations (*n* = 9) about organizing a narrative approach.

**Results:**

Four tensions were identified: (1) steering on predetermined themes vs. emergent themes from experiences; (2) abstracting narratives into broad quality terms vs. preserving the richness of narratives; (3) organizing quality top-down and in silos vs. as a collaborative and dynamic process; and (4) accounting for compliance vs. learning for improvement.

**Discussion and Implications:**

The tensions show that organizing a narrative process goes beyond organizational challenges but requires a paradigm shift in how organizations view and organize quality improvement. It requires a different way of data collection, analysis, and collaboration and emphasizes relationality and learning over individualized accounting. When organizations get stuck between two different quality paradigms, this can lead to several risks. The implications, risks, and potential of narrative approaches are discussed.

## Background and objectives

Whereas long-term care for older persons has historically been characterized by a medicalized approach, the attention has now shifted to person-centered care (Berkovic et al., 2024; Ivanitskaya & Bogner, 2024), with a focus on optimizing the functional ability and well-being of older persons with significant declines in capacity (Pot et al., 2023). To assess person-centered long-term care, organizations currently collect experiences of older persons. This is largely done with standardized questionnaires, such as the Patient Reported Experience Measure, the Consumer Quality Index, or Resident VIEW (Fujisawa & Klazinga, 2017; Tunalilar & White, 2026; Zuidgeest et al., 2012). Themes in these questionnaires are predetermined, leaving limited room to capture the diverse and unique lived experiences of older persons in their daily lives and what these experiences reveal about the quality of care. Instead, quality of care is increasingly seen as processual and relational, which is hard to grasp in standardized measurements (Sion et al., 2020c; Soklaridis et al., 2016). Several scholars and practitioners therefore look for alternative ways to gain insights into how person-centered care from the perspective of older persons and their relatives can be improved.

To gain insight into the quality of care from the perspective of older persons, narrative approaches to quality improvement are increasingly adopted by long-term care organizations, often in relation to the relatives and care professionals of these older persons (Grob et al., 2019; Hsu & McCormack, 2012; Sion et al., 2024). Narrative approaches aim to collect experiences from different perspectives, identify dilemmas and frictions in care with the aim to learn from these narratives and improve the quality of care (Grob et al., 2019; Hsu & McCormack, 2012; Sion et al., 2024). In contrast to questionnaires assessing quality of care, which include predetermined topics and questions, a narrative approach is defined by open questions that focus on the individual’s personal experiences. This approach provides contextual and situational insights into preferences and priorities of involved people as well as making visible possible dilemmas and frictions in quality issues (Miller et al., 2019; Sion et al., 2020a).

Despite an increasing number of research showing how care organizations utilize narratives to improve care from the perspective of older persons, the structural embedding of narrative approaches is organizationally complex and challenging (Kalisvaart et al., 2026). Organizational challenges include issues related to time allocation, unclear process structures and guidance, limited opportunities for relationship building, and insufficient leadership support (Buckley et al., 2018; Scheffelaar et al., 2020; Shura et al., 2011; Sion et al., 2020b). Additionally, there are challenges in the way narratives get handled within organizational contexts. For example, to what extent narratives are abstracted in the process (Scheffelaar et al., 2023). Whereas abstracting is useful to zoom out from an individual narrative to overarching themes, it also can lead to losing useful details of the narrative (Scheffelaar et al., 2023). Another study shows that (individual) “solution-oriented” improvements can be easily made, but that the difficulty lies in looking at the underlying values and frictions in reflection meetings (Rutten et al., 2025). Also, the narrative approaches are often conducted in the context of research projects, which end when researchers withdraw from the care organization after project completion (Kalisvaart et al., 2026).

While various organizational challenges are mentioned in existing studies, they remain understudied, as the main focus is on the narrative approach itself rather than on its organizational embedding. It is therefore unclear how care organizations currently experience the various organizational challenges to embed narrative approaches in new quality improvement routines and policies. Given the different development trajectories of narrative approaches, it is also not clear how care organizations deal with frictions between new narrative approaches for quality improvement and existing standardized approaches to quality improvement, which can still coexist and may clash.

In this study, we want to investigate how care organizations for older persons use a narrative approach and what tensions arise in organizing and embedding this narrative process in existing quality improvement routines and policies. The research question is as follows:

*What organizational tensions arise in the organization and embedding of a narrative approach to improve quality of care, and how do organizations respond to these tensions?*

The Netherlands is a useful research setting for this study, as several narrative approaches have been developed and applied there in recent years (Kalisvaart et al., 2026). Also, Dutch policymakers increasingly stress the importance of qualitatively collecting experiences of care users to get insight in the quality of care (Zorginstituut Nederland, 2023). Therefore, two different Dutch care organizations using a narrative approach for a longer length of time to gain in-depth insights were observed. To gain broader insights, this was paired with interviews, including organizations with varying sizes, types, and levels of experience with narrative approaches.

## Theoretical framework

Embedding a narrative approach entails that it is not only introduced but becomes part of daily routines and organizational structures, norms, and meanings of the care institution. This means that the narrative approach is not only implemented—“a process of putting an innovation, new method or program to use within an organizational setting” (Åhlfeldt et al., 2023, p. 2)—but sustained within the organization over a longer time (Rabin & Brownson, 2018).

In this study, we understand the sustainability of a narrative approach as a process whereby “improvements are maintained, new ways of working become routine, surrounding systems are transformed in support and the innovation may even be developed, over a period of time appropriate to a given situation” (Fleiszer et al., 2015, p. 1495, see also Åhlfeldt et al., 2023). Sustainability is therefore not understood as a static outcome, following a rationalistic, top-down perspective, but as an organizational process in which a new way of working gradually becomes part of existing and changing organizational practices (Chambers et al., 2013; Elg et al., 2015; Greenhalgh et al., 2004). To operationalize sustainability further, we distinguish between “routinization” and “institutionalization” as two separate but related concepts (Moore et al., 2017; Slaghuis et al., 2011).

While routinization concerns the incorporation of the narrative approach into everyday practices, institutionalization concerns its embedding within the broader organizational structures, norms, and meanings (Moore et al., 2017; Slaghuis et al., 2011). First, “Routinization means that through the development of organizational routines a new work method becomes part of everyday activities” (Yin, 1981; Yin et al., 1978, as cited in Slaghuis et al., 2011, p. 2). In this case, the new work method is the narrative approach. Characterized by an open process of collecting and discussing experiences, it requires a different approach to data collection, analysis, and collaboration than traditional ways of working with quality information (Castle & Ferguson, 2010; Donabedian, 1988). For example, data collection is open and not guided by predetermined themes, while analysis focuses on situated experiences and incorporates the perspectives of different people (Sion et al. 2024). Making the narrative approach part of everyday activities therefore requires the development of the right skills (e.g., open interviewing) and tools (e.g., an open format) to support its routinization.

Second, the way a narrative approach is routinized is influenced by the broader organizational structures, norms, and meanings in which it is situated (Urquhart et al., 2020). Institutionalization can be understood as “the gradual adaptation of the organizational context, including structures and processes, to the new work practice” (Jacobs, 2002; Slaghuis et al., 2011, p. 2). At an organizational level, this requires a different understanding of the purpose for which quality information is collected. For example, valuing situational quality information rather than prioritizing comparability. In addition, cooperation and broad participation within the organization seem important for adapting the organizational context to a new work practice (Åhlfeldt et al., 2023; Elg et al., 2015). This may require the quality department or management to take on a more facilitating role in enabling professionals and others involved to jointly collect, interpret, and discuss quality information. This shift may also require changes in organizational structures to support and adapt this participatory way of working, such as organizing meetings with a mix of people.

Together, routinization and institutionalization provide us with a lens for examining how narrative approaches are organized and embedded to improve quality of care and how organizational tensions may arise in this process.

## Research design and methods

### Setting

The long-term care organizations included in this study provide different kind of forms of care, including rehabilitation, day care, home care, and 24-hour care in nursing homes.

Within the Netherlands, quality of care for older persons is guided by laws and regulations, professional standards, and policy guidelines at the national level. During the course of this study, a policy change occurred. In 2024, the existing quality framework was replaced by a Quality Compass (Zorginstituut Nederland, 2021, 2023). The previous framework defined good care in terms of “person-centered care delivery,” “well-being,” “safety,” and “learning and improving together,” and required standardized client experience measures and indicators (Zorginstituut Nederland, 2021). In contrast, the Quality Compass emphasizes collaboration and learning together to improve quality of life with attention to open dialogue and involvement of a person’s network (Zorginstituut Nederland, 2023). Indicators are no longer mandatory, allowing organizations to decide which to report. At the same time, a new quantitative client experience questionnaire was developed, with annual reporting of its outcomes becoming mandatory (Zorginstituut Nederland, 2025). Consequently, participating organizations were operating in a changing policy environment. Some had already stopped reporting mandatory indicators, others piloted the new questionnaire, while others awaited further national decisions before reshaping their own quality trajectories.

### Design and data collection

An exploratory qualitative multiple-method design consisting of ethnographic observations, semi-structured interviews, and document analysis was used (Schwartz-Shea & Yanow, 2012). Data were collected between February 2024 and June 2025. To gain both in-depth and broader insights, two organizations were observed for a longer period—providing detailed insights into the process and temporal dynamics of organizing a narrative approach—and additionally interviews were conducted with nine other organizations on the choices and implications of implementing a narrative approach.

For inclusion, narrative approaches were allowed to range from scientifically developed approaches to those created internally within care organizations. These approaches could also vary as to which people are involved in supporting and executing the approach, how narrative data are collected and analyzed. Additionally, the stage of implementation could differ, including organizations that were in the initial phase of adopting a narrative approach, those with more established experience, and those that had previously experimented with such approaches but had discontinued their use.

Organizations were purposively selected to achieve variation in size and experience with narrative practices (maximum variation sampling) (Patton, 2015). For in-depth insight, one small care organization already working with narratives and one large-size organization willing to learn from experiences of their clients were included. These organizations were recruited via the network of the research team. The narrative approach, including preparation for collecting experiences, collection of experiences, analysis, and learning from these experiences, was observed. During these observations, fieldnotes were made focusing on the stories shared; decisions made about use of stories; design of the process; and the introduced topics, interactions, and emotions during discussions. The fieldnotes were worked into an observation report shortly after the observation. Experiences of people involved in organizing and supporting the narrative process were collected by informal conversations during observations and sometimes more formally by using semi-structured interviews (*n* = 3). Also, relevant documentation, such as reports of experiences of involved people, was collected. Furthermore, the preliminary analysis of the collected data was member-checked with quality advisors in one organization and managers in the other. In addition to the member check, sharing interpretations of the data provided a possibility to the organizations to learn from insights for altering their narrative process (Schwartz-Shea & Yanow, 2012). These meetings were recorded and included in the analysis (*n* = 2).

Additionally, semi-structured interviews were conducted with a wider group of long-term care organizations to get a broader understanding of the use of narrative approaches. These organizations were recruited via a knowledge platform, subsidized by the national government, which offers an overview of sixteen narrative approaches (Waardigheid & Trots, 2023). In addition, via a research network about narrative approaches, contact was sought with people involved in coordinating or executing one of these narrative approaches. Via both routes, contact was sought with 15 people. Not all responded, and in some cases, respondents indicated that no one was still available who could provide information about the narrative approach. Ultimately, 10 (online) semi-structured interviews (*n* = 10) were conducted with one to four participants. Respondents with varied occupational positions and roles were included: quality advisors (*n* = 5), people collecting experiences (*n* = 3), directors (*n* = 3), location managers (*n* = 2), consultants (*n* = 2), and a researcher (*n* = 1). The interviews were semi-structured around four themes regarding: the organization of the narrative approach, the reason to use a narrative approach, how the narrative approach is used for quality improvement, and how a narrative approach plays a role in accountability.

In total, this resulted in 11 organizations with 8 types of narrative approaches. For an overview of the included organizations, their stage of using a narrative approach, the size of the care organization, and what data collection methods were executed and with whom, see Table 1.

**Table 1:** Overview of the included organizations, their narrative approach, and which data collection methods were used.

Organization^a^	Narrative approach	Stage of narrative approach	Size of care organization^b^	Data collection method	With whom
Org. A	Own approach	>3 years in use	1 care home(s)	*In depth*: observation (21 hr), interviews (*n* = 3), recorded meeting (1 hr), documents	Two directors, four professionals story committee, people with dementia, care professionals, volunteers, and relatives
Org. B	Own approach	Started and in use	17 care home(s)	*In depth: o*bservation (43 hr), recorded meetings (12 hr), documents	Director, three quality advisors, facilitators of quality assessment, location manager, older persons (with dementia), professionals, and relatives
Org. C	Caring conversations (Sion et al., 2020a, 2020b)	>5 years	14 care home(s)	Interview (*n* = 1), documents	Two quality advisors (*n* = 2)
Org. D	Images of quality	>12 years in use	8 care home(s)^c^	Interview (*n* = 1), documents	Two directors, one secretary of the foundation, quality advisor/coordinator narrative approach (*n* = 4)
Org. E	Client experience conversations	>3 years in use	61 care home(s)	Interview (*n* = 2), documents	Professional using approach (*n* = 1), Quality advisor (*n* = 1)
Org. F	If you ask me, than… (Heerings et al., 2022)	Starting (tested, training supervisors)	6 care home(s)	Interview (*n* = 1), observation (1 hr), documents	Consultant (*n* = 1), researcher and organization advisor (*n* = 2)
Org. G	Caring conversations (Sion et al., 2020a, 2020b) and Together people-oriented (SAM)-methodology	Starting (training interviewers)	9 care home(s)	Interview (*n* = 1), documents	Quality advisor (*n* = 1)
Org. H	Experiences central (Huijg, 2019)	Stopped	4 care home(s)	Interview (*n* = 1), documents	Location manager and quality advisor (*n* = 2)
Org. I	Experiences central (Huijg, 2019)	Incidentally used^d^	18 care home(s)	Interview (*n* = 1), documents	Location manager (*n* = 1)
Org. J	Story as a quality instrument (Scheffelaar et al., 2021)	Stopped	26 care home(s)	Interview (*n* = 1), documents	Professional using approach (*n* = 1)
Org. K	Story as a quality instrument (Scheffelaar et al., 2021)	Stopped	13 care home(s)	Interview (*n* = 1), documents	Older person using approach (*n* = 1)

a Organizations A and B were observed for a longer period of time; Organizations C–K were interviewed about the narrative approach.

b The size of care organizations is displayed with the amount of care home(s) mentioned on the websites of the care organization, often care organizations also offer home care, day care, or rehabilitation.

c Number of care homes of care organization for older persons is used; the narrative approach is part of a foundation within the Netherlands with six care organizations in total.

d Translated into an “activity-book,” which is part of the quality toolbox of the care organization, teams could choose to use this method (not a central part of quality processes).

### Data analysis

Audiotaped interviews were transcribed verbatim. A thematic analysis was performed with the collected observation reports, transcripts, and documents, using the data analysis software Atlas.ti (version 22). For the first cycle of coding, attribute, descriptive, emotion, and process codes were used (Saldaña, 2013). Attribute coding was used to capture contextual aspects of the data (e.g., participant role, organizational context), while descriptive coding was applied to identify features of narrative practices within the transcripts (Saldaña, 2013). These included aspects like leader of the process, actors involved in collecting experiences, methods of collecting experiences, and approaches to learning and improvement based on these experiences. The different aspects of the narrative approaches were relevant to get insights into approach variety, which are summarized in Table 2. Furthermore, emotion and process codes were used to gather emotions and characteristics of processes while organizing a narrative approach (Saldaña, 2013). For example, emotion codes could be expressions of frustration, reticence, reassurance, or happiness of participants. Process codes were supporting, prioritizing, or adapting aspects of the narrative approach.

**Table 2:** Overview of the characteristics of the narrative approaches, who is involved, how experiences are collected, analyzed and used for learning.

Organization^a^	Narrative approach	Who is responsible for the narrative process?	How are experiences collected?	Who collected and analyzed experiences?	How is the learning process structured?
Org. A	Own approach	Directors (*n* = 2)	Volunteers, care professionals, and relatives via Google form with two open questions; group conversations with older persons	The directors executed the group conversations; a story committee (relative, care professionals, directors) made the Google Form and analyzed the narratives together	An afternoon with volunteers, care professionals, and relatives to talk about the collected experiences and the dilemmas resulting from the analysis?
Org. B	Own approach	Quality advisors (*n* = 3)	Individual conversations with older persons and their relatives; group conversations with care and domestic professionals, volunteers, and facility workers.	A team (external chair and two professionals from the own organization) collect the experiences and held the group conversation. The external chair analyzed the collected experiences and discussed this with the others.	A 2 hr meeting with care professionals, managers, director, (volunteer), older person, and relative to discuss collected experiences and topics that arose from them.
Org. C	Caring conversations (Sion et al., 2020a, 2020b)	Quality advisors (*n* = 2)	Several triads of experiences are collected from older persons, their relative and care professional.	A (care) professional who is trained in the method and working at another location of the organization; the professional write the individual narratives down; the narratives are analyzed by the quality advisors and location manager who formulate tips and tops.	A meeting with care professionals, the manager, and a quality advisor is planned in which the findings are presented (tips and tops retrieved from the narratives), and the care professionals discuss points of improvement. The outcomes are also discussed at regional meetings and in the yearly report of the organization.
Org. D	Images of quality	Advisor quality and relation (functions as coordinator and process supervisor) (*n* = 2)	Care practices are observed following a certain method, including older persons and care professionals.	A (care) professional, older person, or volunteer from outside the organization who is trained in the method observe care practices (4 × 4 hr) and make a detailed observation report (“a mirror”); the conversation partners read the report and prepare two perspectives, appreciation of what is going well and what could be developed.	A meeting with the team and (external) conversation partners (e.g., from other disciplines or from the municipality), led by a process supervisor. In this conversation, the “mirror” is discussed and improvement points are discussed and secured in follow-up process or year plan.
Org. E	Client experience conversations	Quality advisor (*n* = 1)	An individual conversation with several older persons of a location following a guideline with topics in line with the policy of the organization.	A (care) professional working at another location interviews the older people and makes a report (one A4) about all the conversations and fills the information per conversation in the online survey tool “Analyzer.”	The report is given back to the location, and the location itself determines how to discuss this at the location (e.g., the professional joins the team meeting). Coordinator discusses the outcomes at a management (cluster) level, four times a year. Directors discuss overall outcomes every year in comparison to previous years.
Org. F	If you ask me, than… (Heerings et al., 2022)	Consultant (*n* = 1) and organization advisor (*n* = 1)	Group conversations are held with older persons, relatives, and care professionals separately, followed by group conversation(s) together.	A trainer is supporting the process. Analyzing and discussing experiences is happening within the group discussions. The amount of group conversations depends on the question.	The group discussions that are part of the process are also the meetings in which learning from the experiences is facilitated.
Org. G	Caring conversations (Sion et al., 2020a, 2020b)	Quality advisor (*n* = 1)	Several triads of experiences are collected from older persons, their relative, and care professional.	A (care) professional who is trained in the method collect experiences in the triad; makes an overview/rapport; no information yet about the analysis process.	No information yet about the form of the learning process within this organization.
	Together people-oriented (SAM)-methodology	Quality advisor (*n* = 1)	Observations are held within a living room of the organization, including older persons and care professionals.	A (care) professional who is trained in the method makes observations of which an overview/rapport is made.	The overview is discussed within a team meeting of the location
Org. H	Experiences central (Huijg, 2019)	Researchers (*n* = 2)/location manager (*n* = 1)	Care professionals were asked to write down observations of older persons in relation to the experience with care.	The (care) professional makes observations in daily practice, reports about these observations and alters the life enjoy plan of the person (e.g., alternative version of the care plan, to report not only on medical characteristics of care).	During team meetings, experiences and moments of appreciation with care provision, were shared, sometimes by using a photo or object fitting the resident.
Org. I	Experiences central (Huijg, 2019)	Researchers/location manager (*n* = 1)	Care professionals and relatives were asked to write down observations of older persons in relation to the experience with care. Relatives could also share narratives of experiences.	The (care) professional reports about these observations and alters the life enjoy plan of the person. This could be reported in the Sense maker Application.	During team meetings when discussing residents, gathered narratives were shared, followed by reflection on the narratives (e.g., different methods were developed in the “activity-book”) and discussing if a different course of action is useful.
Org. J	Story as a quality instrument (Scheffelaar et al. 2021)	Researcher/location manager	An individual interview with older persons of a location.	A (care) professional who is trained in the method collected experiences and made a portrait of the person. The portraits were analyzed by other (care) professionals, clients, and/or manager to extract themes.	The extracted themes were discussed, and a location manager could pick up these themes to initiate improvement efforts.
Org. K	Story as a quality instrument (Scheffelaar et al., 2021)	Researcher/location manager	An individual interview with older persons of a location.	A (care) professional who is trained in the method collected experiences and made a portrait of the person. The portraits were analyzed by other (care) professionals, clients, and/or manager to extract themes.	The extracted themes were discussed, and a location manager could pick up these themes to initiate improvement efforts.

a Organizations A and B were observed for a longer period of time; Organizations C–K were interviewed about the narrative approach.

Combining these first codes, overarching themes were formulated and discussed within the research team. Overarching themes included the role of narratives, the role of organizing quality, and the role of accountability. In discussing these overarching themes, the tensions in organizing narrative approaches and the influence of the stage of experience with organizing a narrative approach became apparent. A second cycle of coding was then conducted to analyze how these tensions arise and how organizations respond to them. The tensions were summarized and discussed within the research team. In these discussions, tensions were refined; for example, tensions related to internal and external accountability were merged into one analytical theme. This led to a final set of four themes, as described in the result section.

### Ethical considerations

The research ethics committee within the Erasmus School of Health Policy and Management, Erasmus University Rotterdam, approved the study (ETH2122-0455). All participants were verbally briefed about the study before the interviews and observations were conducted. For the interviews and when observed repeatedly, participants also gave written consent.

## Results

From the analysis of the data, four tensions were identified: (1) steering on predetermined themes vs. emergent themes from experiences; (2) abstracting narratives into broad quality terms vs. preserving the richness of narratives; (3) organizing quality top-down and in silos vs. as a collaborative and dynamic process; and (4) accounting for compliance vs. learning for improvement. These tensions played out differently between different care organizations. Tensions were most apparent in organizations that started off working with a narrative approach while still having an existing quality framework based on measuring and compliance. Organizations that had already worked with narrative approaches for a longer period seem to experience these tensions to a lesser extent (or not at all).

### Steering on predetermined themes vs. emergent themes from experiences

The first tension is between the need to steer toward predetermined quality themes that are considered important by the organization vs. emergent themes from the experiences shared by people involved. When experiencing this tension, three organizations decided to prioritize predetermined themes over narratives of involved people (Org. B, E, K).

A first reason for prioritizing predetermined themes seems to be the concern that information about all quality themes, which are considered relevant by the organization, might otherwise not be captured. In Organization B, the narratives of older persons and their relatives were collected at the beginning of the quality improvement process with the aim of guiding improvement in subsequent dialogues with a mix of professionals. This did not work out due to the organizational pull to also prioritize predetermined quality themes as described within the quality framework of the organization. In this framework, themes like “*autonomy*,” “*teamwork*,” and “*infection and hygiene prevention*” were already predefined as guiding themes for quality improvement. During narrative interviews with older persons and dialogues with a mix of professionals, we observed that questions were not open-ended but already linked to predetermined themes of the quality framework (*Fieldnotes Org B., collecting experiences, and conversation mix professionals*). The quality advisor reflected afterwards, as follows:

*I was worried about this, because we discussed this beforehand. But it was the board that really wanted to. Those building blocks had to be included. And then you see that at a certain point they become leading, leading in the conversation. Which means that the conversation about the clients’ experiences is discussed much less*.

(Reflection meeting Org. B, quality advisor 2)

Because quality themes were predefined, collected narratives slowly disappeared into the background. The facilitator of the narrative approach also mentioned that the organization seems to still “walk two paths,” with on the one hand wanting to collect narratives and on the other hand wanting to gather information about all predetermined themes of the quality framework (*Fieldnotes Org. B, meeting facilitator and researcher*).

A second reason for organizations to steer on predetermined themes was the ability to compare different locations within a care organization. Organization K, an organization that had discontinued using a narrative approach after the researcher withdrew, embraced the principles underlying a narrative approach and adapted it to the needs of their organization. Mixed group conversations were organized to discuss an element of the vision on the quality of care of the organization. For these conversations, the facilitator received a list with topics to address:

*Yes it is, it’s certainly useful to have them [the steering questions] come back. And usually, in that conversation, I keep an eye on the time, and then after 45 minutes I say “gosh what a nice conversation, but I have to check if I’m doing everything I agreed to.” I will make something of it, so I can take a quick peek. “Oh, we haven’t talked about this, yes, can you tell me anything more about that [topic]?” So, then I have to steer it a bit. […] That is useful because of the reporting, and those [reports] are compared with each other and analyzed. So it is nice to be able to compare those questions.*

(Interview Org. K, facilitator)

The steering questions were introduced by the quality team to be able to obtain a comparable image about the quality of care across different locations. This showed that not older persons, their relatives, or professionals determined the themes—which could be different for each location—but the need of the organization to compare the different locations.

In organizations that had been working with the approach for a longer period, this tension was less visible or absent, as reflected in the themes of the narratives (Org. A, C, D). The guiding question for gathering experiences of older persons or other involved people was openly formulated and remained open and was used to compare locations based on the themes discussed by them (e.g., do several locations mention the same topics instead of asking each location about the same topics?).

### Abstracting narratives into broad quality terms vs. preserving the richness of narratives

The second tension is situated between the need for abstracting narratives into broad quality terms and preserving the richness of the narratives themselves. When narratives are translated rather quickly into broad quality terms, details of the narratives, which could provide useful insights into underlying organizational processes or frictions/dilemmas in the provision of person-centered care, are likely to get lost.

In Organization B, we observed that topics raised in narratives of older persons were quickly grouped under the broad term of “autonomy.” After the conversations with older persons and their relatives, central themes across all narratives were sought by the facilitator of the narrative approach. This was summarized as follows in the report:

Autonomy is a theme that comes up in almost all conversations. Clients express a need for more agency, for example, when setting the table during meals and discussing shower times during the week. Clients value being taken seriously, for example, when they indicate they want to get out of bed on time in the morning. They also understand that this is not always possible.

(Document Org. B, excerpt from summary of collected experiences made by facilitator)

In this summary, examples of the broad quality term “autonomy” are described, which relate in this case to wanting to be able to influence or direct one’s own life, but the underlying organizational processes or frictions/dilemmas remain undiscussed. For example, what organizational routines are in place that make supporting the agency hard, or does the agency of clients conflict with other values professionals strive for? In the rest of the quality process, the broad quality term “autonomy” was introduced without providing details of the narratives (*Fieldnotes Org. B, conversation mix professionals*). In the final session, a delegation of the organization (professionals, older persons, relatives, managers, and directors) came together to formulate learning goals based on the themes identified in the collected narratives. The theme “autonomy” is discussed as follows:

The facilitator says, “I want to think the same way about ‘autonomy’. All the conversations have touched on freedom of choice, self-reliance, and how to increase it. Just take a minute to think about what comes to mind and then we will discuss it centrally.”

An older person asks about autonomy, what is meant by it? “Do you mean that you give them [residents] the chance to discover it yourself and do it yourself, the greater the reward for the person, and most importantly, if it goes wrong, look at it from the positive side. Is that what you mean by it?” The facilitator responds, “it’s about self-reliance, what can the residents still do themselves, what do they want to try themselves, how can they be independent?”

(Fieldnotes Org. B, learning session with delegation of the organization)

This observation shows that just introducing the theme “autonomy” results in confusion and results in a dialogue in which meaning making of this theme has to occur again. This shows that if a narrative is too quickly translated into broad quality terms, the richness of the narratives to provide contextual, situational, and personal insights about this quality issue within this specific organization gets lost.

A first reason why organizations prioritized abstracting narratives into broad quality terms was the requirement of a report with predetermined quality themes (Org. B, E). The facilitators of the narrative approach were expected to write a report following these predetermined themes, which led to translation of narratives to fit these themes at an early stage. In a later pilot round, one of the facilitators deliberately left some predetermined themes open. The organization was not yet sure how to deal with this in future quality processes (*Fieldnotes Org. B, evaluation pilot 2–4*). Within organization A, the idea and format of a report about the findings was only thought of after the analysis, so no preexisting lay-out of a report was available, allowing themes to emerge more openly from the narratives (*Fieldnotes Org. A, story-committee meeting 4*).

A second reason for abstracting narratives into broad quality terms instead of preserving the richness of narratives could be the skills and training of professionals to interpret and analyze the narratives. In scientifically developed approaches, training was often provided for conducting the narrative approach. Through training, skills were developed such as withholding judgment (Org. H), asking open and nonsuggestive questions (Org. E), or asking follow-up questions about what a person has shared (Org. J). Training also covered how professionals can guide the process of discussing narratives together and listen to each other (Org. H). These training activities often combined theory and practice, including a lot of practice with interviews and/or working methods (Org. J, H). Training to develop these skills was not provided within Organization B, and also not in Organization A. However, the analysis process in Organization A was organized together to discuss common threads. In this process, directors actively steered toward discussing dilemmas in care provision rather than concrete improvement points:

Director 2: “then it’s good to think about the story day, for example the dilemma with the vision, you can talk about that.” Director 1: “yes in relation to basic care, that was also shared last year, to explore [that dilemma] together; the strength of that afternoon is that everyone is present.” […] Director 2: “the goal of the day is that we discuss the most important themes, not everything.” The relative nods in agreement and director 2 continues: “that we can show through the report, the point has been made.” “And awareness that it’s not pleasant,” says a care professional. […] Director 2: “thus tensions in basic care. Maybe the importance of activities vs. too many stimuli.” The relative: “that’s a good one”.

(Fieldnotes Org. A, story-committee meeting 4)

By actively steering and translating the themes into dilemmas rather than specific quality terms and supporting these dilemmas with excerpts of narratives of involved people, their richness was preserved.

### Organizing quality top-down, and in silos vs. as a collaborative and dynamic process

A third tension is situated between the need to organize quality processes top-down and in silos vs. as a collaborative and dynamic process. When quality improvement is seen as a collaborative and dynamic process, space for shared ownership and mutual trust arose, and quality instruments could be more flexibly adjusted. In contrast, when quality improvement is viewed as a primary responsibility of the quality department, then shared ownership, mutual trust, and flexibly adjusting quality instruments were less present.

Shared ownership of the narrative process could be seen in organizations where several perspectives were not only included in the narrative approach itself but also in preparing, executing, and evaluating the narrative process. For example, jointly formulating the questions, jointly identifying a suitable quality approach, and jointly conducting the analysis (e.g., a story committee (Org. A), a work group to set up a new quality process (Org. G), and mixed groups to perform the analysis (Org. A, J, K)). This was different in Organization B, where we observed that organizing the quality process was initially seen as a matter of the quality department (“team quality”), resulting in organizing and developing the instruments in the narrative process in isolation of the facilitators of the narrative approach itself. During the course of experimenting with the narrative approach, this resulted in problems for the facilitators of the narrative approach, and the quality team started to cooperate more with the facilitators of the narrative process in altering the instruments and process (*Fieldnotes Org. B, evaluation pilot 2–4*). This shows that such collaboration is necessary to make the narrative approach work.

Some larger organizations had difficulty in developing trusting relationships at multiple levels of the organization to engage in collaborative quality improvement. For example, the (lack of) trust between the board of directors and the quality team, but also from a quality team to the facilitators of a narrative approach and to the professionals of a care location (*Reflection meeting Org. B*). In some organizations that had been using a narrative approach for a longer period, there was a shift from the organization’s responsibility to visit each location every two years to the locations’ responsibility to participate in a quality cycle based on their needs:

*I come as a process facilitator. But I only come as a team says “gosh [name coordinator], would you like to come by?” And that was different until three years ago. You were simply appointed back then, but we have made a conscious effort to change that. And, fortunately, it all goes smoothly. […] You truly want ownership from a team. And when a team is allocated, it is sort of–well if you are exaggerating, it has to come from above. And now that there is intrinsic motivation, you may strengthen it, at least in my experience, by focusing on the learning objective during the initial meeting. As a result, they [the professionals] are already eager to get started, and you have clearly set the expectations [for the process]*.

(Interview Org. D, coordinator narrative approach)

Moving the responsibility to teams for taking part in a narrative approach also demonstrates trust in the locations to determine themselves what and when to work on quality issues. Other organizations were also planning to move responsibility to care teams after using a narrative approach for several years (Org. G, I).

To enable a transition from organizing quality as top-down and in silos to a collaborative and dynamic process, it was important to have space to experiment. Within Organization B, we observed that actively labeling the process as “experimental learning” was used to alter the quality approach during the process:

*If we don’t learn anything from this, why did we do this? Do you know? We said that as we’re going to use what we’ve learned from this experiment; we can take that with us. Just at the next [round], so we can make a start and then evaluate it. Because otherwise, I just think it would be a bit of a waste*.

(Reflection meeting Org. B, quality advisor 2, Org. B)

Whereas formerly team quality was used to finish a quality approach at the start of a new quality process and to wait for formal approval of the board, they now had more space to experiment during the course of developing a new quality process, making it more dynamic and collaborative with facilitators of the narrative approach.

### Accountability for compliance vs. learning for improvement

The fourth tension is situated between the need to account for compliance vs. learning for improvement, both within an organization and in interaction with external stakeholders. Internally, when different people within the care organization are used to monitor and measure to what extent care practices comply with quality standards, this inhibited the development of a learning environment.

To get used to the new way of working in which quality measures are focused on learning instead of accounting, it requires experience with the narrative process. The participating professionals often expected to be controlled, whereas these processes were more about appreciating what goes well and learning how to improve things (Interview Org. G). Within Organization B, who started working with a narrative approach, a professional explained:

The coordinator says, it is still a kind of test moment, a measurement moment. Is it good enough? Are we performing well enough? While this is more: have a conversation with each other, listen to each other. It is not about good or bad, but could it be different?

(Fieldnotes Org. B, evaluation location and quality advisor)

The previous way of working was received as a measuring moment, which was underscored by more organizations (Org. B, C, G, F).

A clear vision about the use of the narrative approach and actively fostering dialogue and supporting the learning process is useful to be able to open up as a care professional and not become defensive or only saying what is not working well (*Interview Org. F*). As also described within an organization working for a long period with a narrative approach:

*When asked what it actually contributes to… It is about pausing, looking in the mirror at, you know, the work of everyday practice, reflecting, giving meaning to quality [of care], recognizing and improving what is going well. So, pause, reflect, gather understanding, and then find areas for improvement. And the process is really active, collaborative learning. You also assume that there is enough experience in-house to complement one another*.

(Interview Org. D, coordinator)

The organizations working for a longer period with a narrative approach stressed the goal of learning and reflecting together most strongly (Org. A, D).

Externally, when working with narratives and focusing on quality as a learning practice, organizations are negotiating with external parties (e.g., local authorities, health insurers, or inspectors) to be able to provide a learning environment for their professionals. These external parties are often more comfortable with or require (again) numbers or indicators for accountability, which could influence learning for improvement negatively. This resulted in changing or stopping auditing practices within included organizations and negotiation with external stakeholders about what accountability should look like.

Various organizations that have developed their own (narrative) quality system decided to stop their participation in external auditing (Org. B, D) or have chosen an external auditor/certification that fits their more narrative way of looking at quality (Org. G, H). For example, Organization G has chosen to change their auditing service, which no longer aligns with their vision of learning about quality in daily practice:

*Because there were quite a few rules, quite tightly regulated, and we specifically wanted our employees to be able to work on quality in their daily practice; we wanted to meet the residents’ needs. So we started looking, okay, which other certification agencies are being embraced by other long-term care organizations?* (Interview Org. G, quality advisor)

In addition to switching to another auditing agency, this organization also engages in discussions with the external auditor if the auditor approached the audit too much as “ticking the boxes” (*Interview Org. G*). This shift to another kind of auditing service shows a way of dealing with the tension and to move to fostering an environment of learning instead of only complying. It also shows that auditing agencies are moving from using only “check-boxes” to more dialogue-based auditing, which requires other skills and adjustments of involved auditors (*Interview Org. H*).

However, discontinuing external auditing is not always easy, especially when municipalities insist on requiring external certification to obtain the municipal tender, as the following director explains:

*Sometimes it is really bizarre. I mean, we tender for domestic help from all municipalities, right? A municipality that has been satisfied with us for years is now simply sending back a new tender, because we do not have a specific certificate or whatever. And actually, it is a waste of time of course. And if I disagree, I have two days to respond. Well, okay, I think I can describe what we do, why I believe we provide good quality, and what our support is. Then you will figure it out, but before you know it, you will have lost your entire tender because you did not check a box. Those are absolutely the hiccups of our time, I believe.*

(Interview Org. D, director 1)

Whether an organization can and/or dares to make the choice to stop with external auditing therefore also depends on what payers and regulators expect from care organizations and the work organizations do to negotiate with such actors.

The same difficulty applies for interactions between care organizations and health insurers, i.e., how to give insight in working on quality in a narrative way. The director mentioned the rationalized practices of health insurers, which often focus on numbers:

Director 2: *“Well, in itself, it works; it truly touches their hearts. I honestly believe they all truly understand the meaning, especially when they participate [in a narrative approach]. However, in their world […] it is incredibly difficult to explain, so what you are experiencing is unique on a systemic and energetic level. Yes, and you don’t squeeze that into an Excel file when reporting, as is common in the highly rationalized practice of the health insurers.”* Board secretary: *“Yes. People seek control, don’t they? Some level of control, oversight, and understanding into where all those resources are going? And then trust, as the only issue, is still a bit insufficient.”*

(Interview Org. D, director 2 and board secretary)

When organizations were unable to get affirmation from external parties that narrative approaches were approved, they were sometimes hesitant to keep using them (*Interview Org. H*). Meanwhile, Organization D also negotiated with the Health and Youth Care Inspectorate (HYCI) to alter the investigation process to be able to facilitate learning from a serious incident using a narrative approach rather than the standard assessment (*Interview Org. D, director 1*). These examples show the work organizations must do to provide an environment for learning for improvement. This involves changing or stopping with auditing practices and negotiating with diverse external stakeholders.

## Discussion and implications

We investigated which organizational tensions arise when organizing and embedding narrative approaches to improve quality of care and how organizations respond to these tensions. Four tensions were found: (1) steering on predetermined themes vs. emergent themes from experiences; (2) abstracting narratives into broad quality terms vs. preserving the richness of narratives; (3) organizing quality top-down and in silos vs. as a collaborative and dynamic process; and (4) accounting for compliance vs. learning for improvement. The presence of these tensions and how organizations respond to them varied between included organizations.

The results show that routinization of a narrative approach requires a different way of data collection (e.g., open, based on themes of care users); analysis (e.g., focus on situated experiences instead of downplaying *n* = 1); and collaboration (e.g., between different layers of the organization, focusing on learning). For this, the right skills (e.g., open interview technique, leading group discussions, inductive analysis) and tools (e.g., open interview questions, reporting without predetermined themes) were needed to enact these changes in daily practice. However, how the narrative approach was enacted in daily practice was also shaped by the institutional context in which these tools and skills were used. Institutionalization of the narrative approach depended, for example, on the supportive position of the quality department or management to enable people to collectively collect, analyze, and discuss experiences instead of top-down quality audits. Also, existing norms and understandings of what constitutes valuable quality information (e.g., situational knowledge vs. comparability) shaped how the approach was interpreted and applied in practice.

Organizing and embedding a narrative process goes thus beyond merely addressing *organizational challenges*, but requires a paradigm shift in how organizations view and organize quality improvement (Greenhalgh & Papoutsi, 2018; Kuhn, 1962). This paradigm shift entails a new way of looking at quality of care, which emphasizes relationality and learning over individualized accounting. This implies that implementation efforts should not only provide technical skills training for collecting and analyzing narratives but should also create opportunities for the gradual development of a new shared understanding of narrative quality information. Therefore, attention should not only be paid to the organizational conditions like supportive leadership and sufficient time (Kalisvaart et al., 2026) but also to how underlying intentions, values, and instruments shape what is considered valuable quality information.

When organizations get stuck between two different quality paradigms, this can lead to several risks. First, narratives can be used in a tokenistic way: i.e., while narratives are collected, they are not actually used to learn (Carr & Ann, 2011; Fricker, 2007). This is the case when organizations prioritize the quality themes of the organization instead of the people determining the themes. Second, when abstracting narratives too quickly into overarching themes, relevant details can get lost (Scheffelaar et al., 2023), or older persons may not recognize their own narratives and perspectives any longer, leading to confusion and a lack of dialogue. Third, narratives may be used for incidental problem solving by having only attention for details instead of deeper reflections on the meaning of situations or possible dilemmas (Rutten et al., 2025). Organizations then do not reflect on organizational work routines, practices, and agreements that influence, for example, experienced autonomy or loneliness (e.g., morning rounds, privacy restrictions). Fourth, expecting objectivity, comparability, and measurability within a narrative process can undermine the uniqueness of narratives of older persons and others involved (Reinders & Nazarowa, 2020). The focus can also shift back to “ticking the box” rather than dialogues in order to learn together.

To overcome these risks and realize the full potential of narrative approaches, it is important for organizations to reflect on the dominant quality view present within their organization. First, directors and managers could reflect on how their intentions to use narrative approaches relate to existing ideas and work routines with regard to quality improvement. The tensions identified in this study could help them to anticipate hurdles and required commitment. By saying this, we do not suggest that quantitative quality information could not still play a role in quality improvement. Quantitative information may remain useful for assessing clearly defined aspects of quality, tracking trends, or as signals to further investigate certain quality issues. However, understanding more complex and relational quality issues requires qualitative information that provides insight into people’s experiences and the context in which care is provided. Second, it is useful to provide space and trust for quality teams or advisors to experiment with new ways of collecting information about quality of care. In that way, quality advisors are able to adapt and respond to challenges in using a narrative approach (Devi et al., 2021; Vosters et al., 2026). When organizations work together on different layers of the organization this could lead to more trust, enabling a more dynamic and collaborative narrative process (Devi et al., 2021). Third, providing time for taking part in quality improvement projects and providing training to develop relevant skills—like open interview techniques, analyzing techniques, and leading group discussions—is helpful to enable deeper reflections for learning (van Delft et al., 2023; Rutten et al., 2025). Besides these “technical” skills to conduct the method itself, development of expertise and shared understanding of the purpose of quality information and the underlying view of quality is important. When organizations invest time in a culture of learning and skill development, more responsibility can be moved to the level of the team. It could also lead to less incidental problem solving and more structural improvements at the level of the organization.

The current Dutch care policy for older people encourages dialogue and learning (Zorginstituut Nederland, 2023). This seems challenging in practice for care organizations who operate in a field with various external parties who require compliance instead of learning to improve (Gunningham, 2012; Møller & Hill, 2021). For external parties, such as payers, accreditation organizations, and regulators, knowledge about narrative approaches is needed to be able to ask the right questions and to challenge organizations when narratives are used in a tokenistic way. Additionally, other regulatory strategies to make this shift from compliance to internal learning are needed (Pot, 2022; Pot et al., 2024; RVS, 2023). When external parties and long-term care organizations work with the same intention to learn to improve, this could further help to make a paradigm shift to person-centered care from the perspectives of the care users themselves.

### Limitations

Although the in-depth-cases were followed for a longer period, observing actual quality improvements was outside the scope of this study. These processes often take longer than was possible in this study. For future studies, it could be relevant to conduct longitudinal studies on narrative quality improvement to get more insight into the impact these processes have on person-centered care. Furthermore, the participants interviewed were diverse and could not always answer questions about the view of external parties in relation to their narrative way of working. However, those who shared their experiences named the same kind of external parties that influenced their choices around working on quality in a narrative way. Future studies could investigate the institutional work external parties, in relation to care organization, have to do to alter their practices fitting this new paradigm. Reflexive regulation, focused on stimulating learning with different stakeholder about a complex societal issue, could be useful but further research is needed (Pot, 2022; Pot et al., 2023).

## Data Availability

The data, analytic methods, or materials are not available to other researchers for replication purposes due to ethical restrictions. There was no preregistration of this study.
